# A new model based on improved VGG16 for corn weed identification

**DOI:** 10.3389/fpls.2023.1205151

**Published:** 2023-07-07

**Authors:** Le Yang, Shuang Xu, XiaoYun Yu, HuiBin Long, HuanHuan Zhang, YingWen Zhu

**Affiliations:** ^1^ School of Computer and Information Engineering, Jiangxi Agricultural University, Nanchang, China; ^2^ Software College, Jiangxi Agricultural University, Nanchang, China

**Keywords:** attention mechanism, corn weed, deep convolutional neural network, global average pooling, Leaky ReLU

## Abstract

Weeds remain one of the most important factors affecting the yield and quality of corn in modern agricultural production. To use deep convolutional neural networks to accurately, efficiently, and losslessly identify weeds in corn fields, a new corn weed identification model, SE-VGG16, is proposed. The SE-VGG16 model uses VGG16 as the basis and adds the SE attention mechanism to realize that the network automatically focuses on useful parts and allocates limited information processing resources to important parts. Then the 3 × 3 convolutional kernels in the first block are reduced to 1 × 1 convolutional kernels, and the ReLU activation function is replaced by Leaky ReLU to perform feature extraction while dimensionality reduction. Finally, it is replaced by a global average pooling layer for the fully connected layer of VGG16, and the output is performed by softmax. The experimental results verify that the SE-VGG16 model classifies corn weeds superiorly to other classical and advanced multiscale models with an average accuracy of 99.67%, which is more than the 97.75% of the original VGG16 model. Based on the three evaluation indices of precision rate, recall rate, and F1, it was concluded that SE-VGG16 has good robustness, high stability, and a high recognition rate, and the network model can be used to accurately identify weeds in corn fields, which can provide an effective solution for weed control in corn fields in practical applications.

## 1 Introduction

China is a large agricultural country with a population of 1.4 billion, and the development of agriculture affects all aspects of life. Weeds can significantly affect crop yield and quality, and weed control is a laborious and tedious task. Herbicide spraying and manual weeding are the most common methods of weed control; however, they are not desirable from an economic or environmental point of view, and manual weeding is more costly and less efficient. With the modernization of our countryside, combining computers with agricultural production has become an inevitable trend (Sujaritha et al., 2017; Jin et al., 2021; Rong et al., 2022). Weed identification using deep learning (Jin et al., 2012; Xu et al., 2021) can not only improve the weed identification rate and rationalize the use of weed control methods; but also make efficient use of herbicides to protect the environment. As innovations in deep learning theory and hardware conditions continue to develop, people can construct deeper network models to extract more features (Ye et al., 2019; Wagle and Harikrishnan, 2021), and an increasing number of network models are being constructed for use in various aspects of agricultural production (Chakraborty et al., 2021). Deep learning has been widely applied in recent years, especially in smart agriculture fields, such as pest and disease detection (Mique and Palaoag, 2018; Liu et al., 2022; Wu et al., 2022), plant and fruit recognition (Jaiganesh et al., 2020; Bongulwar Deepali, 2021), and crop and weed detection and classification (Pando et al., 2018; Jin et al., 2021). Image recognition technology has long been used for weed recognition applications (Jiang et al., 2020). In 2019, Jiang et al. (Jiang, 2019) proposed a new model to identify field weeds by adding transfer learning to VGG16. The final model achieved good results on 12 weed images with 91.08% accuracy in the validation set. Liang et al. (Liang, 2019) subsequently constructed a new network model for weed identification by adding transfer learning to Inceptionv3 network, and the final training accuracy was over 99%. Subsequently, Barbedo (2019) proposed a method to increase the image dataset. Multiple diseases in different plants were considered in this experiment. Using the proposed method, the accuracy increased by 12%. In 2020, Fu et al. (2020) proposed a new model based on a VGG network to identify weeds in fields. Using the Kaggle image dataset, the detection accuracy of field weeds reached over 98%, and in real fields, the accuracy reached 80%. In 2022, Shundong et al (Fang et al., 2022). proposed the HCA-MFFNet for maize leaf disease identification. To validate the feasibility and effectiveness of the model in complex environments, it was compared with existing methods and the average detection accuracy of the model was 97.75%. In 2022, Subeesh et al. (2022) constructed a deep learning method to detect pepper weeds and achieved 98.5% accuracy. Later, Najmeh et al. (Razfar et al., 2022) proposed a weed identification model to improve the efficiency of weed detection in soybean plantation forests using several network models and three custom network models for comparison. The custom CNN architecture exhibited a detection accuracy of 97.7%. In 2023, Zhang et al. (2023) proposed a new model for tomato leaf disease detection by introducing an asymptotic non-local mean algorithm (ANLM) and a multi-channel Automated Oriented Recursive Attention Network (M-AORANet) to extract rich disease features. Experimental results on 7,493 images showed that the recognition accuracy of M-AORANet reached 96.47%.

The LeakyReLU activation function is currently used in a wide range of fields and has been shown to be effective in improving the accuracy of the models. In 2022, Yang et al. (2023) proposed a RE-GoogLeNet network model to identify rice leaf diseases. By replacing the ReLU activation function with the LeakyReLU activation function, RE-GoogLeNet exhibited better classification performance for rice leaf diseases than the other models, with an average accuracy of 99.58%. The SE attention mechanism is also widely used in various networks and has been shown to help models extract image features and improve the network performance. In 2013, an improved network for rock recognition based on the SE attention mechanism was proposed (Huang et al., 2023), yielding an accuracy of 93.2%. Owing to the redundancy of parameters in the fully connected layer, some recent high-performance network models use global averaging pools rather than FCs to fuse learned depth features (Zhao, 2017). In view of these advantages, these methods are used in the proposed model. Starting from the theme of accurate weed control in the field, this study used common weeds in corn fields as the main research objects. Through several groups of controlled experiments, an efficient and stable detection model SE-VGG16 with high detection accuracy was constructed, and the SE-VGG16 model was applied to weed detection of other species with the aim of constructing a weed detection model with the ability to generalize weed control in the agricultural production process. The SE-VGG16 model proposed in this study makes the following five major contributions to existing models:

1. A new model, SE-VGG16, was proposed for identifying weeds in agricultural crops.
2. The number of parameters in the model is reduced from 70M to 15M by replacing the 3 × 3 convolutional kernel in the first block of VGG16 with a 1 × 1 sized convolutional kernel.
3. The SE attention mechanism is added to the VGG16 model to obtain more important feature information using a weight matrix that gives different weights to different positions of the image from the perspective of the channel domain.
4. ReLU is replaced by Leaky ReLU to obtain more image features and reduce the sparsity of ReLU.
5. Using a global average pooling layer to replace two fully connected layers can better unify the global spatial information corresponding to the last convolutional layer of the category and feature map, thereby integrating the global spatial information and improving the robustness of the model.

## 2 Materials and methods

### 2.1 Image collection

**Figure 1:**
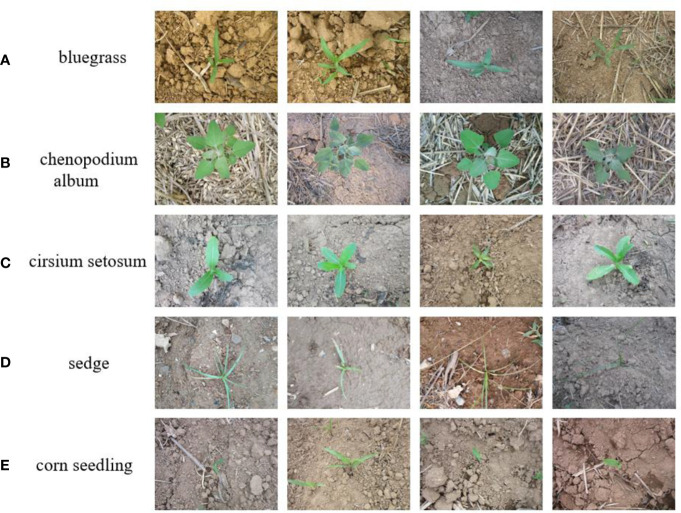
Images of some samples, **(A–E)** of Bluegrass, Chenopodium album, Cirsium setosum, Sedge and corn seedling, respectively.

Images of corn seedlings and weeds were collected from Gitee (https://gitee.com/Monster7/weed-datase/tree/master/) via the Internet, and the corn weed dataset was taken from fields of corn seedlings in their natural environment. A Canon PowerShot SX600 HS camera was used, with the camera pointing vertically towards the ground to reduce the effect of sunlight reflections. After expert identification and manual screening, a total of 6,000 images were obtained, including images of one corn seedling and four corn weed species, with the weed categories of Bluegrass, Chenopodium album, Cirsium setosum, and Sedge. Some examples of images are shown in **Figure 1**, and the data for different image categories in the dataset are shown in **Table 1**.

**Table 1:** Corn seedling and weed data set.

Type	Number
bluegrass	1,200
chenopodium album	1,200
cirsium setosum	1,200
sedge	1,200
corn seedling	1,200

### 2.2 Model building

#### 2.2.1 VGG16

**Figure 2:**
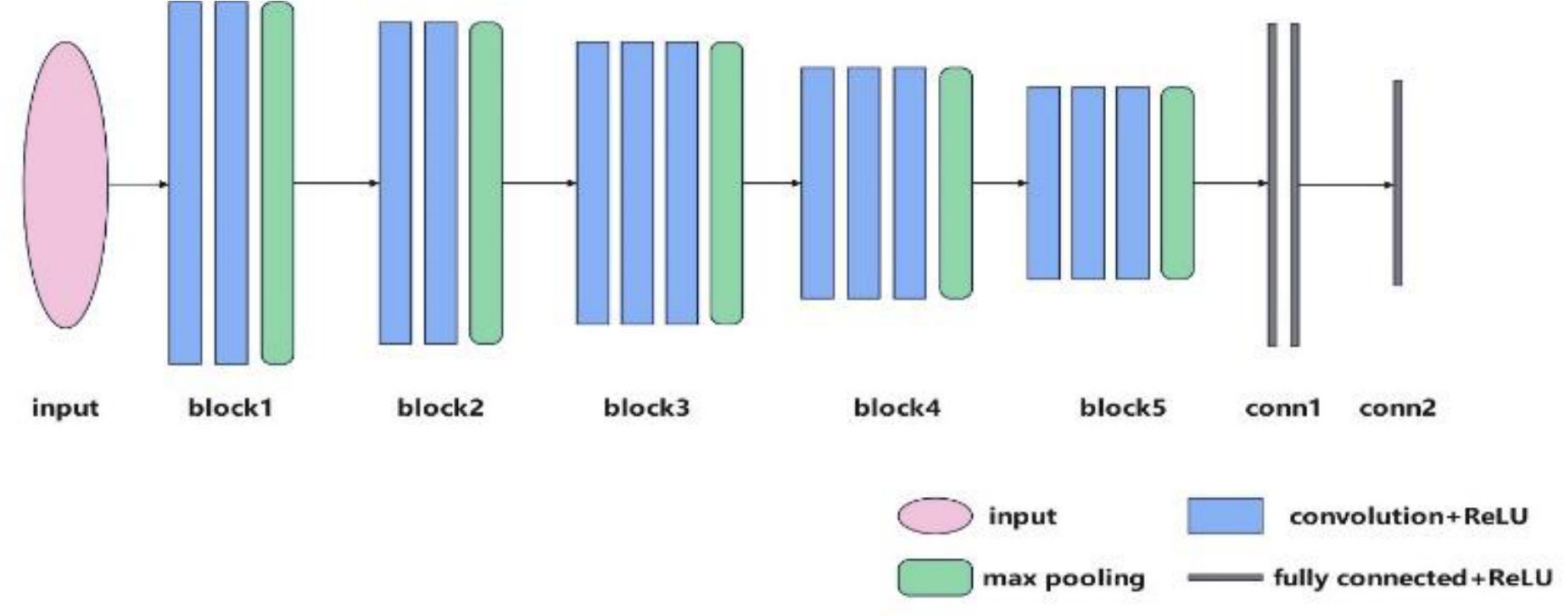
VGG16 structure diagram.


VGGNet (Simonyan and Zisserman, 2014) achieved the second place in ImageNet image classification in 2014, with VGG16 being a particularly high-performance network in VGGNet. VGG16 means that there are 16 layers containing parameters in the model, here they are divided into five blocks and a section of fully connected layers, the structure of the model is shown in **Figure 2**, there are two convolutional layers in block1 and block2, three convolutional layers in block3, block4, and block5, the size is 3 × 3 for the convolution kernel, and the ReLU activation function is used after each convolution, each block has a maximum pooling layer at the end of the block, and the size is 2 × 2 for the pooling kernel. The final segment of the VGG16 network consisted of three fully connected layers, and the final output was obtained using the softmax function. The mathematical expression for the softmax function is given by Equation 1. The implication of softmax is that instead of uniquely determining a maximum value, each output classification is assigned a probability value, indicating the likelihood of belonging to each category, where Z_i_ is the output value of the *i*th node and C is the number of output nodes, that is, the number of categories in the classification. The Softmax function transforms the output of multiple categories into a probability distribution ranging from [0, 1].

(1) $$Softma\text{x}\left(z_{i}\right)=\frac{e^{z_{i}}}{\sum_{c=1}^{C}e^{z_{c}}}\;\;$$

#### 2.2.2 SENet

Attention mechanisms (Bahdanau et al., 2014; Vaswani et al., 2017) aim to achieve a network that automatically focuses on useful parts and allocates limited information processing resources to important parts. Attention mechanisms include soft and hard, and soft attention mechanisms include spatial (Jaderberg et al., 2015), channel (Hu et al., 2019), and mixed domain (Fe et al., 2017) attention mechanisms.

**Figure 3:**
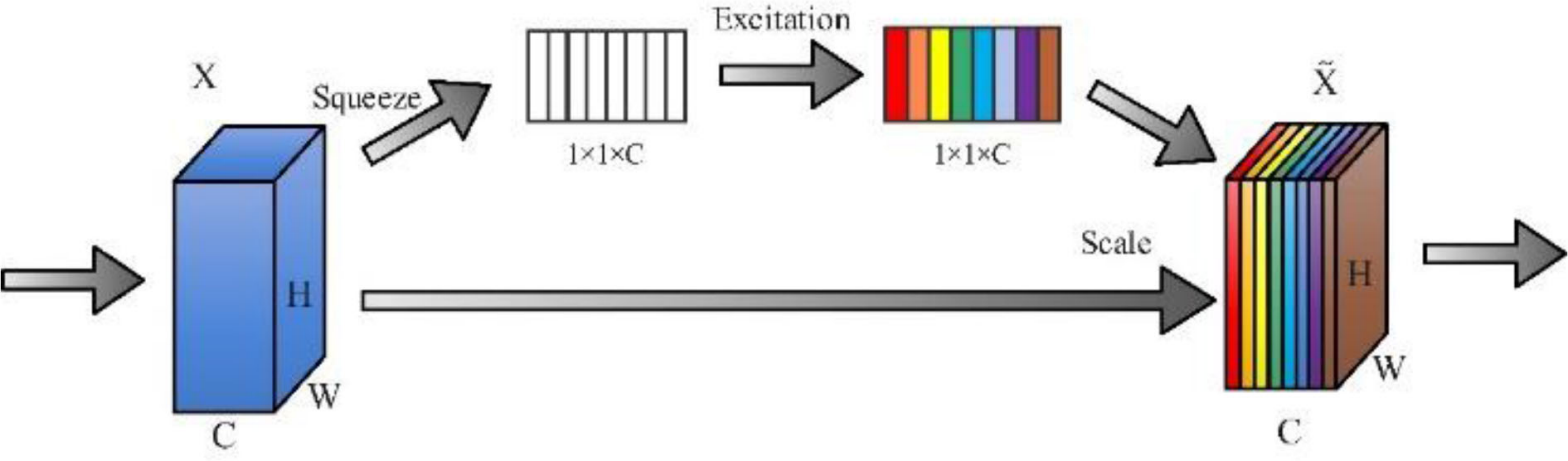
SENet structure diagram.

SENet (Hu et al., 2019) was proposed in 2017 and won the image classification task in the ImageNet 2017 competition, with the structure shown in **Figure 3**. It comprises two main components, squeezing and excitation. In the Squeeze operation, the global average pooling of C feature maps of size H × W is performed, and the feature maps of C × H × W are compressed into one-dimensional feature maps of size 1 × 1 × C, i.e., the global information of one-dimensional H × W is obtained with a global perceptual field. In the excitation operation, 1 × 1 × C one-dimensional features obtained from the squeeze operation are added to the fully connected layer, and the significance for each channel is obtained by the parameter W, which generates weights for each feature channel to show the correlation between each channel. Finally, the weights of the excitation output are multiplied with the previous features by channel-by-channel multiplication to achieve recalibration of the original features.

#### 2.2.3 Global average pooling

**Figure 4:**
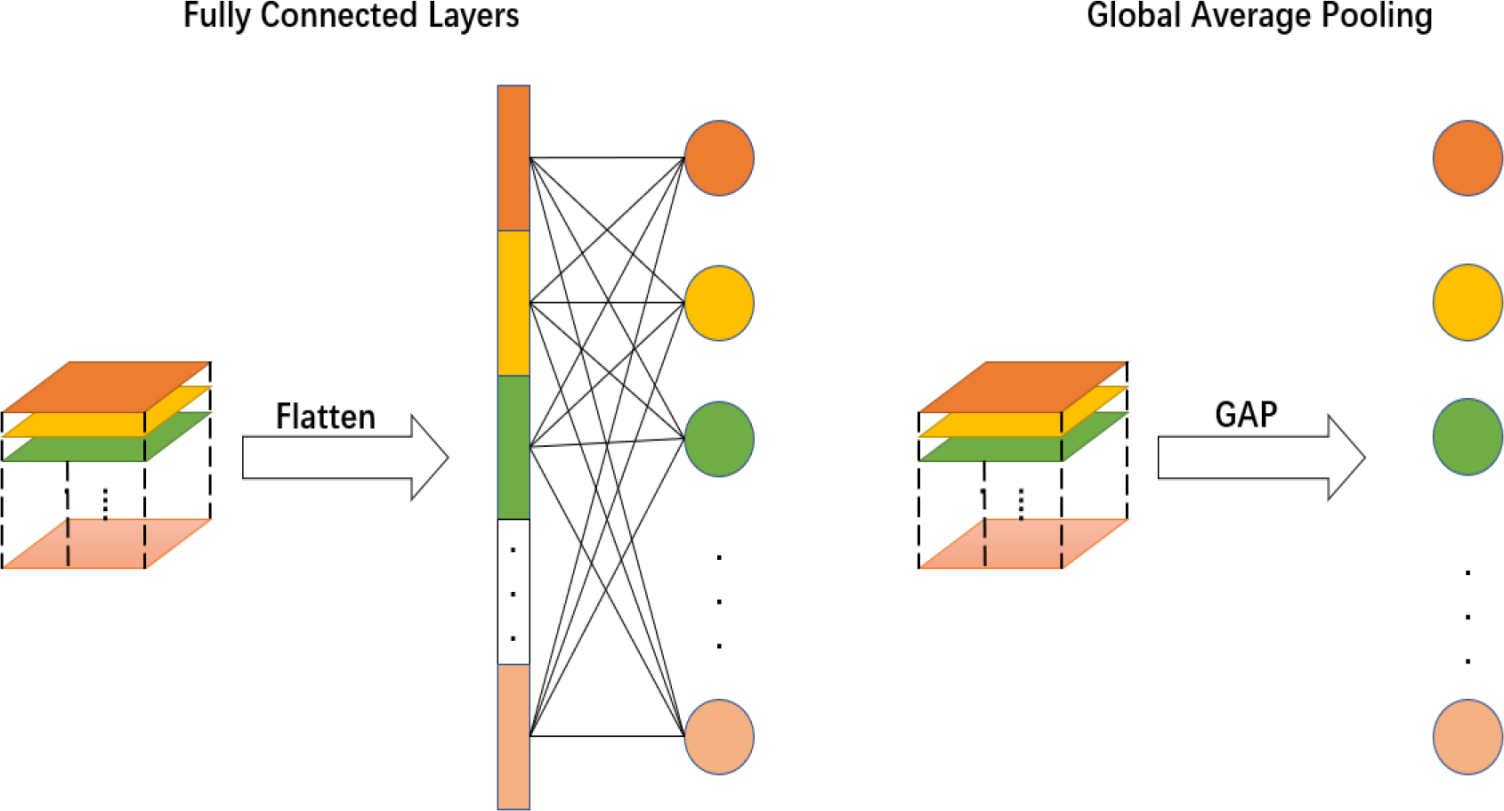
Comparison of fully connected layer and global average pooling layer.

The global average pooling layer (Lin et al., 2013) was first proposed in Network in Network in 2013 and is widely used in large convolutional neural networks. Traditional neural networks often have one or two fully connected layers; however, the number of parameters used is very large, which tends to cause overfitting. Global average pooling is an important component of the network, and the specific implementation is to calculate an average value for all pixels of the feature map of each channel of the output, to obtain a feature vector with a dimension equal to the number of categories, and then directly input to the softmax layer, which can achieve the effect of dimensionality reduction, thus reducing overfitting and improving the recognition accuracy of the network. In addition, by adding global averaging pooling, the model can have a global perceptual field so that the underlying network can also use global information to achieve better results. A comparative diagram of these results is shown in **Figure 4**.

#### 2.2.4 Leaky ReLU activation function

**Figure 5:**
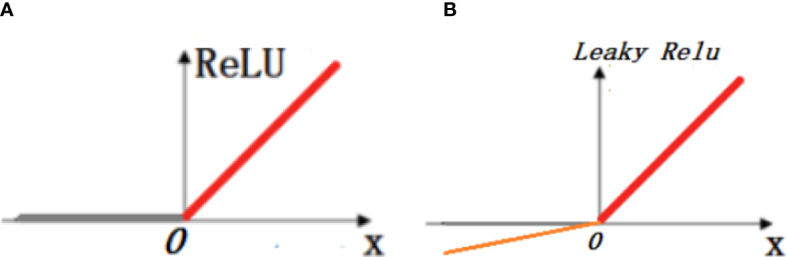
Plot of ReLU and Leaky ReLU activation functions. In the figure, **(A)** shows the linear representation of the ReLU activation function and **(B)** shows the linear representation of the Leaky ReLU activation function.


The ReLU activation function (Liang and Xu, 2021) is a modified linear unit with a mathematical expression, as shown in Equation 2, and input x. If x >0, its gradient is positive and can be used for weight updating, defined as the active state; if x<0, its gradient is 0, the weights cannot be updated and the neuron is in a non-learning state, defined as the inactive state. To solve this problem, a linear unit function with leakage correction, that is, the Leaky ReLU function, is introduced. Compared with the ReLU function, the Leaky ReLU function retains a very small constant a in the negative axis; therefore when the input information is less than 0, the information is not completely lost and is retained accordingly, solving the problem that the neurons are not activated in the negative interval of the ReLU activation function. The mathematical expression of the Leaky ReLU function is shown in Equation 3, and **Figure 5** shows the plots of the ReLU and Leaky ReLU activation functions.

(2) $$\text{ReLU}\left(\text{x}\right)=\text{max}\left(0,\text{x}\right)=\{\begin{array}{l} 0,\;\text{if}\;\text{x }\leq \;0\;\;\left(\text{Inactive}\;\text{state}\right)\\ \text{x},\;\text{if}\;\text{x }>\;0\;\;\left(\text{Active}\;\text{state}\right) \end{array}$$

(3) $$\text{LeakyReLU}\left(\text{x}\right)=\text{max}\left(\text{ax},\text{x}\right)=\{\begin{array}{c} \text{ax},\;\;\;\;\;\text{if}\;\text{x }<\;0\\ \text{x},\;\;\;\;\;\;\text{if}\;\text{x }\geq \;0 \end{array}$$

#### 2.2.5 SE-VGG16

**Figure 6:**
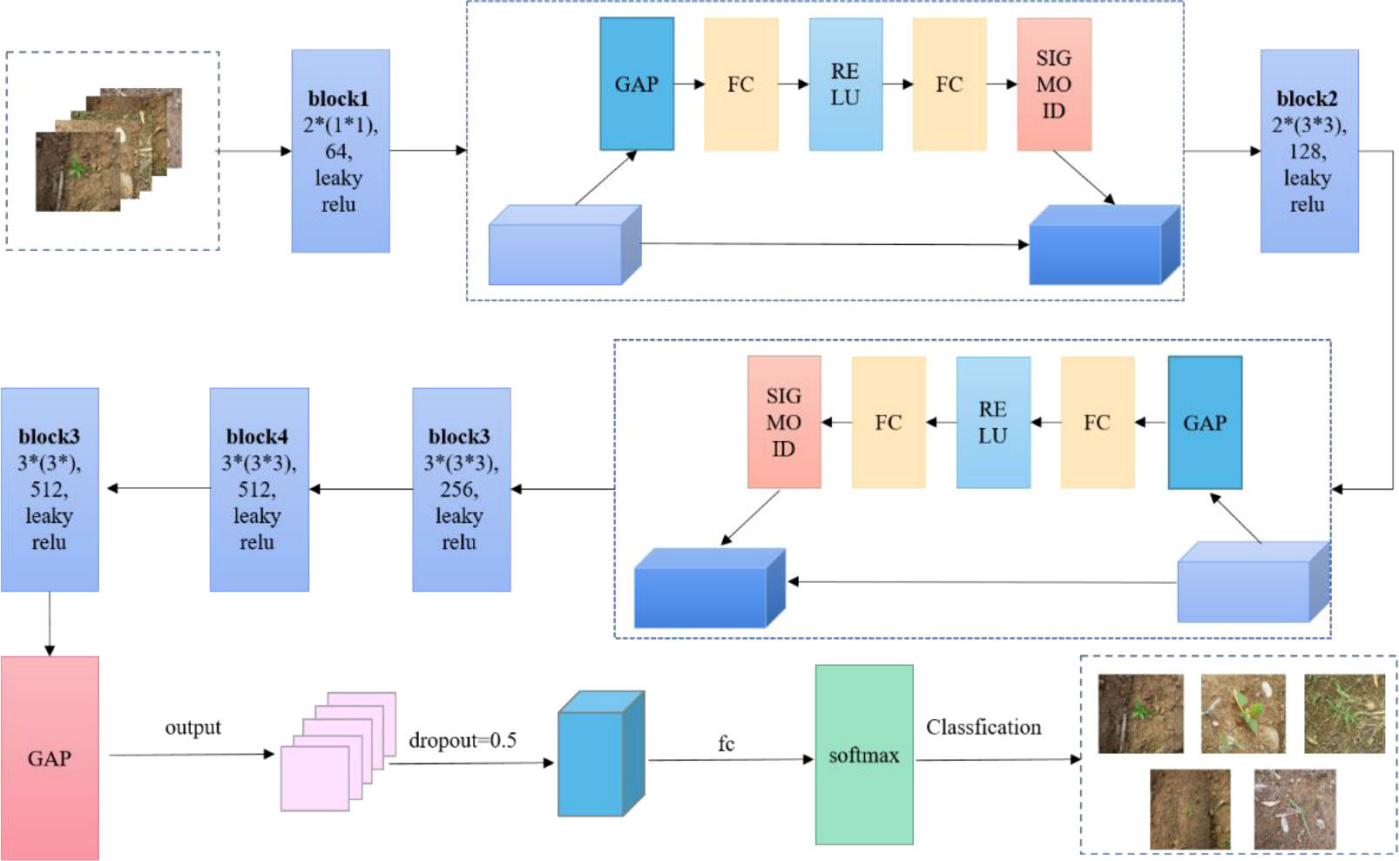
SE-VGG16 construction diagram.

In this study, based on VGG16, the SE attention mechanism was added after the first and second blocks, respectively, to enable the network to automatically focus on the useful parts and allocate the limited information processing resources to the important parts; then the 3 × 3 convolutional kernels in the first block were reduced to 1 × 1 convolutional kernels, and 1 × 1 convolution was used to reduce the number of channels in the deep neural network to reduce the number of channels without reducing the computational complexity without degrading the model performance, replacing ReLU with the Leaky ReLU activation function. Leaky ReLU can improve the accuracy of the model in some cases, as it allows some negative outputs that may contain useful information, replacing the fully connected layer in VGG16 with global average pooling to reduce the risk of overfitting the model and speed up the training speed, and finally outputting with softmax, whose structure is shown in **Figure 6**.

## 3 Experimental results

The experiments were conducted on a Alienware computer (Dell, Inc, Round Rock, USA) configured with AMD Ryzen 7 5800H; 3.20 GHz, 24.0 GB RAM; NVIDIA GeForce RTX 3060 graphics; Windows 10 64-bit operating system. CUDA version 11.2, Cudnn version 8.1, Tensorflow 2.5.0, Python 3.7. The software used mainly included OpenCV image-processing software, with the parameters listed in **Table 2**.

**Table 2:** Parameter settings.

Parameter	Value
Batch size	16
Optimizier	Adam
Learning rate	0.0001
Epochs	200
Activation	Leaky ReLU
Dropout rate	0.5

### 3.1 Experimental results and design

**Figure 7:**
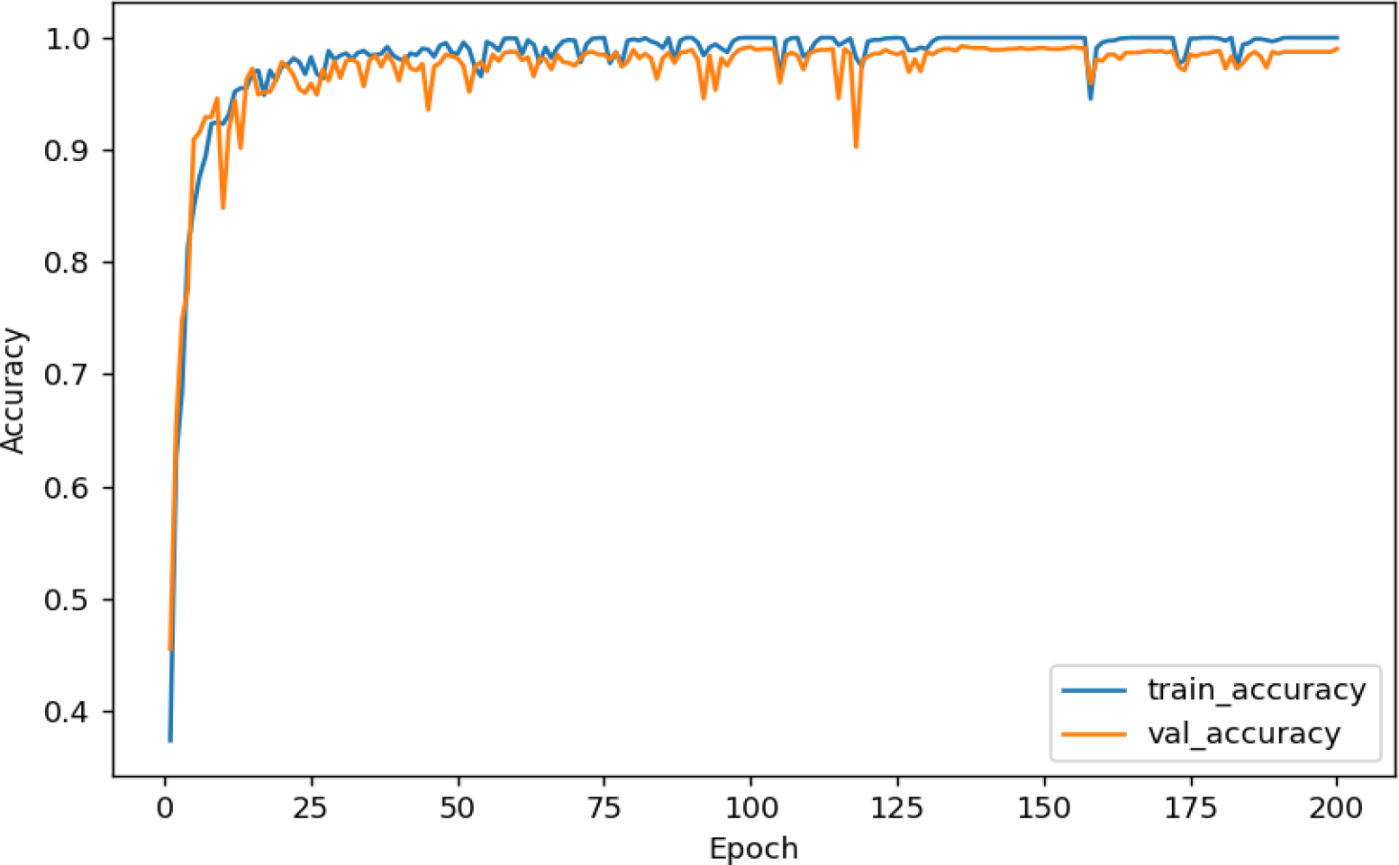
SE-VGG16 training set and validation set accuracy curves.

**Figure 8:**
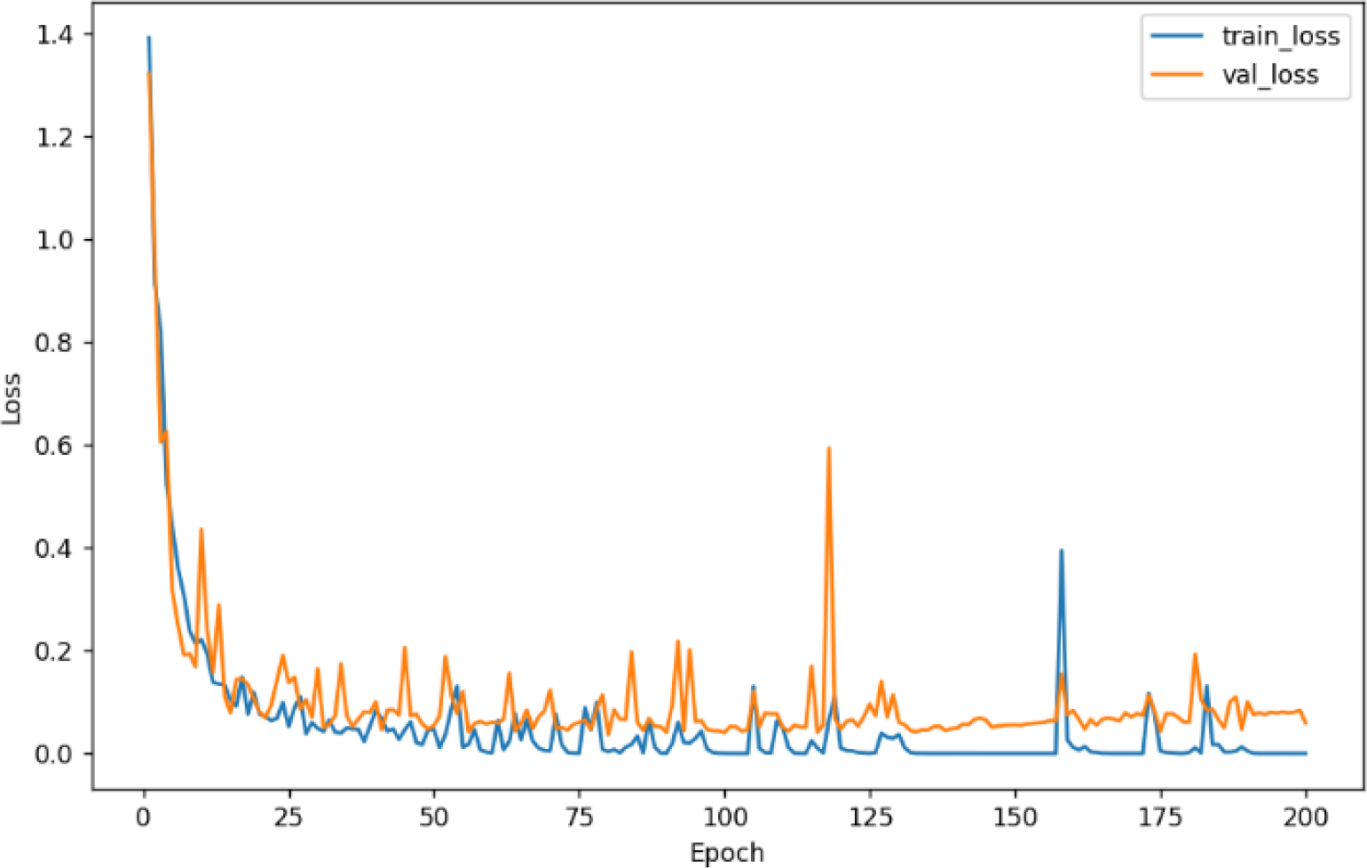
SE-VGG16 training set and validation set loss rate curves.

For this experiment, the dataset was divided into a training set, validation set, and test set in a ratio of 6:2:2, and then the images were input to a uniform size of 224 × 224. **Figure 7** shows the accuracy curve and **Figure 8** shows the loss rate curve of SE-VGG16 training. The figure shows that the model started to converge after 25 iterations. As the number of iterations increases, the two curves gradually fit together, and the difference between the two accuracies is close to 0, indicating that the model has reached the fit state and achieves a good training effect.

To further validate the generalization ability of SE-VGG16, other experiments were conducted in this study, namely, different activation function comparison experiments, different attention mechanism result comparison experiments, comparison tests of multiple SE modules added to the model, ablation experiments, model validation experiments in other datasets, and comparison of results with other models.

### 3.2 Evaluation metrics

In this study, the Precision, Recall, Accuracy, and F1 were used to measure the performance of SE-VGG16 in identifying corn weeds, calculated as follows:


(4) $$Precision=\frac{TP}{TP\;+\;FP}$$

(5) $$\;Recall=\frac{TP}{TP\;+\;FN}\;$$

(6) $$Accuracy=\frac{TP\;+\;TN}{TP\;+\;FN\;+\;FP\;+\;TN}\;$$

(7) $$F1=\;\frac{2TF}{2TP\;+\;FP\;+\;FN}$$

TP denotes predicting a positive sample that is positive, i.e., a correct prediction; FP denotes predicting a negative sample that is positive, i.e., an incorrect prediction; FN denotes predicting a positive sample that is negative, i.e., an incorrect prediction; and TN denotes predicting a negative sample that is negative, i.e., a correct prediction.

### 3.3 Comparison of the results of different activation functions

**Figure 9:**
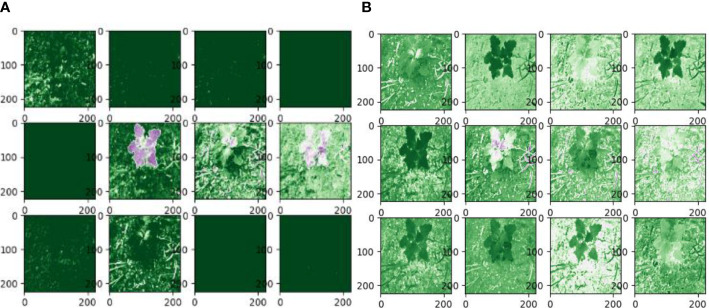
Partial convolutional layer visualization. In the figure, **(A)** is the feature layer visualization of the ReLU activation function, and **(B)** is the feature layer visualization of the Leaky ReLU activation function.

**Figure 10:**
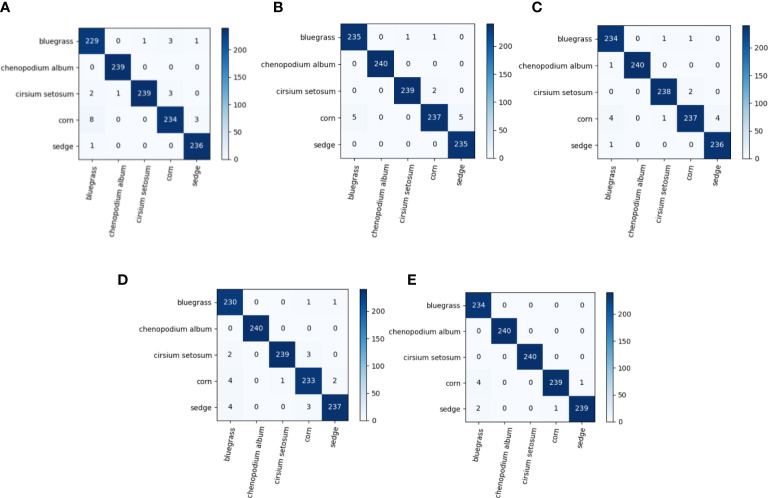
**(A–E)** show the confusion matrix for the ReLU, Elu, Swish, Selu, and LeakyReLU activation functions, in that order.

The original VGG16 model uses the ReLU activation function, but the ReLU activation function can lead to “necrosis” in some neurons (Xu et al., 2020). This means that the neuron stops responding to any input during training and permanently outputs a 0. This is because, if the value of the input is less than 0, the gradient is 0, and the neuron does not update its weights during backpropagation and continues to output 0 without learning any useful features from the data. To avoid the above problem, this study additionally selected four common activation functions to investigate their effects on network performance: Leaky ReLU, Swish, Elu, and Selu; the results are shown in **Table 3**. By comparing the evaluated values of the four activation functions, it was found that the accuracy of the model using the other three activation functions was higher than that of the model using ReLU, while Leaky ReLU worked best with an accuracy of 99.33%. Finally, we visualize the feature layers of ReLU and Leaky ReLU separately, as shown in **Figure 9**, which shows that Leaky ReLU has better performance for images. **Figure 10** shows the confusion matrix for each experimental group.

**Table 3:** Comparison of different activation functions.

Type	ReLU	Elu	Swish	Selu	Leaky ReLU
Batch size	16	16	16	16	16
Epochs	200	200	200	200	200
Acc%	97.75	98.83	98.75	98.25	99.33
Pre%	97.78	98.88	98.78	98.26	99.36
Rec%	97.76	98.84	98.76	98.26	99.34
F1%	97.77	98.86	98.77	98.26	99.35

### 3.4 Comparison of the results of different attention mechanisms

The three commonly used attention mechanisms are CBAM, ECA, and SE. In this study, the three attention mechanisms are added to the VGG16 network separately, keeping each parameter the same and using Leaky ReLU for the activation function, and the values are displayed in **Table 4**. Experiments showed that the SE attention mechanism performed well in this network and was best for classifying corn seed-lings with weeds.

**Table 4:** Comparison of different attention mechanisms.

	Eps	Acc%	Pre%	Rec%	F1%
Add CBAM	200	98.67	98.68	98.66	98.67
Add ECA	200	99.17	99.16	99.18	99.17
Add SE	200	99.33	99.36	99.34	99.35

### 3.5 Comparison test of multiple SE modules added to the model

To further verify the effect of the attention mechanism in the model, we added one to five SE modules to the model, and the results are presented in **Table 5**. The best results were achieved when the SE module was added to the first and second blocks of the model, with an accuracy of 99.67% and almost no change in the size of the model.

**Table 5:** Comparison test of multiple SE modules added to the model.

	Eps	Acc%	Pre%	Rec%	F1%
1 SE module	200	99.33	99.36	99.34	99.35
2 SE module	200	99.67	99.68	99.68	99.68
3 SE module	200	99.58	99.60	99.60	99.60
4 SE module	200	99.41	99.44	99.44	99.44
5 SE module	200	99.25	99.26	99.28	99.27

### 3.6 Ablation experiment

**Figure 11:**
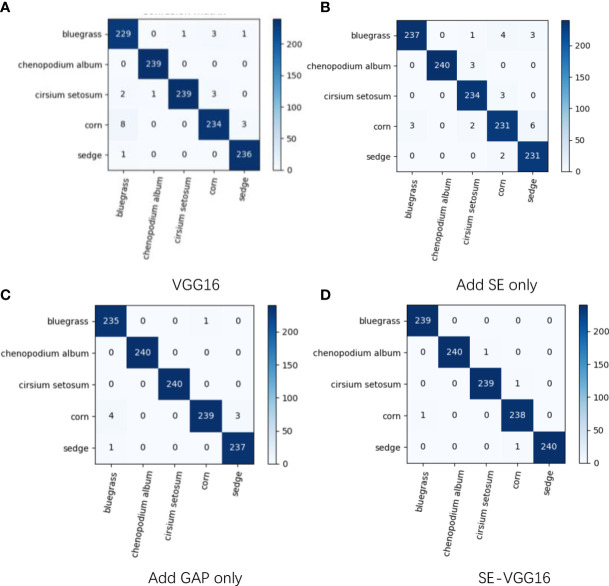
Confusion matrix comparison chart. The figure shows **(A)** the confusion matrix of the VGG16 recognition results, **(B)** the confusion matrix of the model recognition results when only the SE module is added, **(C)** the confusion matrix of the recognition results when only the global average pooling layer model is added, and **(D)** the confusion matrix of the SE-VGG16 model recognition results.

To verify the improved effect of the model, this study trains each classification network under the same condition, which is the network with only SE added, the network with only global average pooling added, the network with SE and global average pooling added, and the VGG16 network. The obtained results are shown in **Table 6**. These data also revealed that the network using SE with global average pooling was the best for classifying corn seedlings and weeds with an accuracy of 99.67%. **Figure 11** displays the confusion matrix plots for the above four networks, which show that SE-VGG16 successfully recognized most of the sample images for each type, and the superiority of SE-VGG16 is again certified in terms of performance.

**Table 6:** Comparison of results of ablation experiments.

	VGG16	Add SE only	Add GAP only	SE-VGG16
Accuracy%	97.75	97.75	99.25	99.67
Precision%	97.78	97.76	99.28	99.68
Recall%	97.76	97.74	99.26	99.68
F1%	97.77	97.75	99.27	99.68

### 3.7 Model validation experiments on other datasets

To validate the generalization ability of SE-VGG16, we obtained datasets of soybean seedlings and weeds from the internet. Typically, convolutional neural networks require many images for learning, and given the existing conditions, a larger number of images cannot be collected. The existing dataset is small and has a large gap with ImageNet. To prevent overfitting, the original dataset was expanded in this study, and the expanded dataset included 1,000 images of soybean seedlings and 1,000 images of each of the two types of soybean weeds: broadleaf and grass, respectively. As in the previous experiments, the dataset was split into a training set, validation set, and test set with a ratio of 6:2:2, and the same parameters as in the previous experiments were kept, and the training was performed for VGG16 and SE-VGG16, respectively; the results are presented in **Table 7**. The results indicated that the accuracy of the SE-VGG16 model reached 99.83% in the soybean dataset, which was higher than the accuracy of the VGG16 model (98.00%), indicating that SE-VGG16 was also applicable to other crops, proving that the model has good generalization ability.

**Table 7:** Table of results for soybean data set.

Models	Type	Pre%	Rec%	F1%	Acc%
VGG16	broadleaf	100	96.50	98.22	98.00
	grass	98.50	98.00	98.25	
	Soybean	95.70	99.50	97.56	
SE-VGG16	broadleaf	100.00	100.00	100.00	99.83
	grass	100.00	99.50	99.75	
	soybean	99.50	100.00	99.75	

### 3.8 Comparison with other model results

To further validate the performance of SE-VGG16, the SE-VGG16 model was compared with other convolutional neural network models, including AlexNet, VGG13, VGG19, EfficientNet, and MobileNetV3, and the results are shown in **Table 8**. Compared with AlexNet, VGG13 and VGG19, SE-VGG16 has a slightly higher accuracy, but the number of parameters is significantly lower, which makes training easier. Compared with lightweight models, such as MobileNetV3, the number of parameters still needs to be further reduced, but the accuracy of SE-VGG16 is 2.59% higher than that of MobileNetV3, further demonstrating the good performance of the SE-VGG16 network.

**Table 8:** Comparison results with other models.

No.	Models	Acc%	Pre%	Rec%	F1%
1	AlexNet	97.17	97.18	97.14	97.16
2	VGG13	97.92	97.90	97.92	97.91
3	VGG19	98.67	98.68	98.68	98.68
4	MobileNetV3	97.08	97.10	97.08	97.07
5	EfficientNet	98.00	98.02	97.98	98.00
6	SE-VGG16	99.67	99.68	99.68	99.68

## 4 Conclusion

To address the low accuracy of the original VGG16 model, the SE attention mechanism is added, which allows the model to focus more on features that are more critical to the classification task while reducing interference from noisy features, thus improving the accuracy and generalization ability of the model. The 3 × 3 convolutional kernels in the first block of VGG16 are then reduced to 1 × 1 convolutional kernels to reduce computation and increase nonlinearity, thereby reducing the number of parameters to 20% and speeding up the computation. The Leaky ReLU activation function was used instead of the ReLU activation function, and feature extraction was performed with reduced dimensionality to achieve a 99.67% accuracy. Compared with the models proposed by other researchers in the literature cited in this paper, our proposed model performs better. However, the number of SE-VGG16 parameters remains high and cannot be easily applied to portable devices. The next step in this research will be to reduce the number of senators in portable devices to automatically track and identify plant seedlings with extensive weed-related knowledge. Combining deep learning methods with weed control in the field improves the efficiency of weed control, saving labor and time costs, while accurate control can also help protect the soil and environment. The research in this paper provides theoretical support for the precise application of herbicides in modern agriculture, as well as for the informatization and intelligence of agriculture. In the future, deep learning combined with agricultural production will be applied to other areas to support the entire process of agricultural production and improve the efficiency of agricultural production.

## Data availability statement

The original contributions presented in the study are included in the article/supplementary material. Further inquiries can be directed to the corresponding author.

## Author contributions

SX and LY contributed to the conception of the study. SX and XY performed the experiment. HZ and HL contributed significantly to analysis and manuscript preparation. YZ and SX performed the data analyses and wrote the manuscript. LY and SX helped perform the analysis with constructive discussions. All authors contributed to the article and approved the submitted version.
